# Activating and inhibitory receptors on natural killer cells in patients with systemic lupus erythematosis-regulation with interleukin-15

**DOI:** 10.1371/journal.pone.0186223

**Published:** 2017-10-12

**Authors:** Syh-Jae Lin, Ming-Ling Kuo, Hsiu-Shan Hsiao, Pei-Tzu Lee, Ji-Yih Chen, Jing-Long Huang

**Affiliations:** 1 Department of Pediatrics, Division of Asthma, Allergy, and Rheumatology, Chang Gung Memorial Hospital, College of Medicine, Chang Gung University Linkou, Taoyuan, Taiwan; 2 Department of Microbiology and Immunology, Graduate Institute of Biomedical Sciences, College of Medicine, Chang Gung University, Taoyuan, Taiwan; 3 Department of Medicine, Division of Allergy, Immunology and Rheumatology, Chang Gung Memorial Hospital, College of Medicine, Chang Gung University Linkou, Taoyuan, Taiwan; Instituto Nacional de Ciencias Medicas y Nutricion Salvador Zubiran, MEXICO

## Abstract

Natural killer (NK) cells may play an important role in the pathogenesis of SLE. Interleukin(IL)-15, an NK-enhancing cytokine, is over-expressed in SLE patients. In the present study, we examined the effect of IL-15 on NK cytotoxicity of SLE patients, and the expression of various activating and inhibitory NK receptors on NK cells from SLE patients in relation to disease activity. We also sought to determine how IL-15 would affect the NK receptor expression on NK cells from SLE patients. PBMCs were collected from 88 SLE patients with inactive disease activity (SLEDAI score<6) and active disease activity (SLEDAI score≥6), 26 age-matched healthy adults were used as controls. PBMC were incubated in the presence or absence of IL-15 (10ng/ml) for eighteen hours. CD3^-^CD56^+^ lymphoctes were gated using flow cytometry and further divided into CD56^dim^ and CD56^bright^ subsets according to the MFI of CD56. We observed that 1. Serum IL-15 was elevated in SLE patients, and higher in active disease than in inactive disease; 2. NK cytotoxicity of SLE patients was deficient compared to controls and showed an impaired response to IL-15 compared to controls; 3.CD69, CD94, NKG2A, NKp30, and CD158b on NK cells from SLE patients were higher than controls, and could be further enhanced by IL-15; 4. NKp46 expression from SLE patients was higher than controls, but down-regulated by IL-15; 5.Deficient NKG2D and NKAT-2 expression were found on NK cells from SLE patients, which were enhanced by IL-15; 6. A unique NKp46^-^ subset and CD158b^+^ subsets were observed in NK cells from SLE patients but not controls. 7. Unlike controls, CD158k on NK cells from SLE patients failed to respond to IL-15. Taken together, we demonstrated the aberrant NCR and iNKR expression on NK cells and their distinct response to IL-15 in SLE patients. As IL-15 predominantly aggravates the aberrant NKR expression found in SLE, IL-15 antagonist may have therapeutic benefits in SLE patients.

## Introduction

Systemic lupus erythematosus (SLE) is a chronic systemic inflammatory autoimmune disease with a wide array of clinical manifestations characterized by the presence of high titers of autoantibodies, elevated circulating immune complexes, and complement deficiency [1, 2]. The etiology and pathogenesis of SLE remained to be elucidated.

Natural killer (NK) cells are CD3^-^CD56^+^ large granular lymphocytes, serve as a first-line defense against viral infection and tumors [3, 4]. According to the CD56 molecule density on NK cell surface, NK cells can be divided into two subsets. CD56^dim^ NK cells express CD16 and are responsible for cytotoxic function. CD56^bright^ NK cells have the capacity to produce abundant cytokines and serve as immunoregulators [5, 6]. Previous studies a have found a decrease in NK cell numbers, impaired NK cytotoxicity and defects of NK differentiation in SLE patients [7–9]. NK cells can serve as a double-edge sword as it may promote the inflammation in SLE by producing interferon-gamma (IFN-_γ)_ which may promote B cell activation and aut0-antibody production [10]. On the other hand, NK cells may ameliorate the inflammation by their ability to kill activated T cells and macrophages. IFN- _γ_ produced by NK cells may also suppress Th17 differentiation [11]. The relationship between NK cell abnormalities and SLE activity was not clearly established and the role of NK cells in the pathogenesis of SLE remains controversial. We have demonstrated the dysfunctional NK and NKT-like cells in SLE patient with regard to CD11b and CD62L expression [12].

NK cell activation is mediated by a series of surface receptors and co-receptors. CD69, a typeII C-lectin membrane receptor which is rapidly induced upon activation, is considered pro-inflammatory as CD69^+^ NK cells were able to induce the TNF-alpha release by monocytes [13]. NKp46 and NKp30 are natural cytotoxicity receptors that constitutively expressed on NK cells and may initiate the immuneattack mediated by NK cells [14, 15]. NKG2D which recognizes several stress-induced ligands expressed by cancerous and virally infected cells were essential for enhancing NK cytotoxicity. [16]

On the other hand, NK cells also expressed several inhibitory receptors that upon activation may suppress their cytotoxic function. CD94, like CD69, a type II membrane protein related to the C-type lectin superfamily. It is covalently associated with the NKG2 family and regulating NK cell function by inhibiting cytotoxicity and promote survival [17]. NKAT-2, with two immunoglobulin domains and a long cytoplasmic tail, belongs to KIR2DL3, and is specific to HLA-C molecule [18]. NKG2A, a highly homologous lectin-like NK cell receptor that form heterodimers with the CD94 molecule, contains immunoreceptor tyrosine-based inhibition motif, is generally consider inhibitory [19]. CD158a (KIR2DL1), a specific receptor for the HLA-C group1 (HLA-Cw2,4,5,and 6) and CD158b (KIR2DL2/L3, KIR2DS2) for the HLA-C group 2 (HLA-Cw1,3,7,and 8), also inhibit target cell lysis by NK cells [20]. CD94, CD158a and CD158b expression were increased on endometrial NK cells from infertile women [21]. CD158k (KIR3DL2), belonging to the family of killer cell immunoglobulin-like receptors (KIR), binds specifically to the HLA-A3 and HLA-A11. CD158k ligation on NK cells inhibits IFNγ and cytotoxic function [22].

Interleukin(IL)-15 is a common gamma-chain signaling cytokine that plays an important role in NK differentiation and survival [23, 24]. Patients with SLE have increased serum levels of IL-15, regardless of disease state [25, 26]. Under the influence of IL-15, the circulating NK cells may be in an activated state in SLE patients.

In the present study, we examined the NK cytotoxic function of SLE patients and explored the expression of various NK receptors on NK cells from SLE patients with different disease activity and their response to IL-15. We also sought to examine the effect of IL-15 on the expression of these NK receptors on CD56^dim^ and CD56^bright^ NK cells from peripheral blood of SLE patients and healthy controls.

## Materials and methods

### Study subject

This study protocols were reviewed and approved by the institutional review board of the institutional of review board of Chang Gung Memory Hospital (Institutional of Review Board No. 102-3935A3 and 102-5508A3).The patient characteristics are summarized in Table 1. We recruited 88 SLE patients with written inform consent from the Out-Patient Clinic of the Division of Rheumatology and Immunology, Department of Internal Medicine, and from Division of Asthma,Allergy and Rheumatology, Department of Pediatrics, Chang Gung Memorial Hospital between January 2014 and June 2016. We also obtained written informed consent from the parents or guardians of the participants under 18 years of age. The diagnosis of SLE fulfills the 1997 American College of Rheumatology classification criteria [27].

**Table 1:** Characteristics of controls and patients with SLE.

Characteristics	Normal (N = 26)	SLE patients	
		Inactive (N = 39)	Active (N = 49)
Sex (Male/Female)	0/26	4/35	4/45
Age	29.7±0.8	34.6±2.2	30.3±1.7
SLEDAI (Median and Range)	NA	2 (0–5)	8 (6–25)
C3, Median (Range, mg/dL)	NA	67.4 (34.8–106)	61.2 (19.3–97.3)
C4, Median (Range, mg/dL)	NA	8.1 (0–32.9)	8.6 (1.72–21.1)
Anti-dsDNA+ (Mean±SEM, WHOunit/ml)	NA	300.5±32.7	354.1±26.1^†^
Nephritis	NA	17.9%	41.7%
Taking regular corticosteroids	NA	90.9%	88.9%
Drug (Mean±SEM, mg/day)	NA	10.4±1.2 mg/day	14.3±1.4 mg/day
CD56^+^CD3^-^ NK cells (%)	8.5±0.8%	7.0±1.1%*	5.9±0.6%**

* p<0.05

** p<0.01, SLE patients (Inactive or Active) compare with normal.

^†^p<0.05, Inactive SLE patients compare with Active SLE patient.

Disease activity was assessed using the SLE Disease Activity Index (SLEDAI) [28], and a score ≥6 defined active disease. According to the cutoff value, 39 patients had inactive SLE while 49 patients had active SLE. Their medication history was carefully recorded and all patients were provided informed consent after approval of the Hospital Ethics Committees. There were no symptoms and signs suggesting infection at the time of blood drawing.

### PBMC incubation

Mononuclear cells (MNCs) were obtained from SLE patients and healthy adult volunteers according to the guidelines of the Human Subjects Protection Committee of the Chang Gung Memorial Hospital and the informed consents obtained from all of the donors. Peripheral blood is collected in sterile tubes containing heparin (20 units/ml of blood) and was processed within 24 hours of collection. MNCs are then separated from heparinized blood using Ficoll-Hypaque density gradient centrifugation. PBMCs were then incubated in RPMI-1640, 10% fetal calf serum in the presence or absence of IL-15 (10ng/ml, Peprotech, Rocky Hill, USA) for eighteen hours.

### NK cytotoxicity assays

Flow cytometric NK cytotoxicity assays were performed as previously described [29]. Briefly, unstimulated or IL-15 treated MNCs (Effectors) and K562 cells (targets, an erythromyelocytic leukemia cell line) were added in duplicate to 12x75 mm round bottom polystyrene tubes at E:T ratios of 12.5:1 and 25:1. Control tubes including only target or effector cells assayed to determine the spontaneous cell death. Tubes were mixed by gently tapping, centrifuged at 200x g for 1 min and incubated at 37°C in 5% CO_2_ for 4 hours. After the incubation, 10μl of CD45-FITC (fluorescein isothiocyanate) (BD Bioscience) were added to each tube. The tubes were then mixed gently and incubated for 20 min on ice. Twenty μls of propidium iodide (PI, Sigma Chemical CO., St. Louis, MO, USA) at 1μg/ml were added to each tube 10–15 min before acquisition. After 4 hours of incubation, percentages of dead K562 cells are calculated from histograms showing fluorescent intensity of PI uptake of K562 cells after subtraction of background cell death.

### Flow cytometric analysis of NK markers

PBMCs were washed in cold PBS with 2% FCS and 0.1% sodium azide and then stained with fluorescein isothiocyanate (FITC)-, R-phycoerythrin (PE) or Allophycocyanin (APC)-conjugated mouse anti-human monoclonal antibodies including anti-CD3/CD16^+^CD56 (FITC/APC), CD69, CD94, NKAT-2, NKG2D, NKp46, NKp30, NKG2A,CD158a, CD158b, and CD158k (PE) (BD Biosciences) from Becton-Dickinson (Worldwide Inc., Taiwan Branch, Taiwan) for flow cytometric analysis.

The fluorescent staining was analyzed on a FACS Calibur (BD Biosciences) flow cytometer. Electronic gates were set to enable analysis of the fluorescence of the viable cell population according to FSC/SSC histograms following anti-CD3/ CD16+CD56 staining. The percentage of cells stained with each monoclonal antibody was determined by comparing each histogram with one from control cells stained with FITC-, PE or APC-labeled isotype control monoclonal antibodies. The lymphocyte population was gated first to identify CD3-positive and CD3-negative lymphocyte populations for the subsequent analysis of the expression patterns of CD56. According to the mean fluorescence intensity of CD56, CD56^+^CD3^+^ NK cells were further divided by fluorescent intensity of CD56 staining in to 2 groups: CD56^dim^ (more than 80%) NK cells and CD56^bright^ NK cells.

### Cytokine assays

Serum levels of IL-15 were quantified by enzyme-linked immunosorbent assay (ELISA) (Quantikine, R & D Systems, Minneapolis, MN, USA) according to the manufacturer's instructions. The lower limit of detection of this assay was 3.9 pg/ml.

### Statistical analysis

The Wilcoxon signed rank test was applied for analysis of the difference of responses before and after IL-15 treatment. SLE patient and healthy control data were compared between groups using the non-parametrical Mann–Whitney U-test. (calculated by SPSS 17.0 software). The data are presented as means ± standard error of mean (SEM). Groups being compared were considered significantly different if *p* value was less than 0.05.

## Results

### Patient characteristics and percentages of NK cells in active and inactive disease

The characteristics of the SLE patient population are shown in Table 1. Patients were predominantly female, age between 15–68 years old and had an average disease duration of 7.8±0.6 years. SLE patient with active disease tend to have more renal involvement, lower C3, C4, and high ds-DNA. SLE patient had decreased percentages of CD56^+^CD3^−^ NK cells (p = 0.007), more so when disease activity is high.(patients with active disease vs. patients with inactive disease, p = 0.045)

### Serum IL-15 is elevated in SLE patients

**Fig 1:**
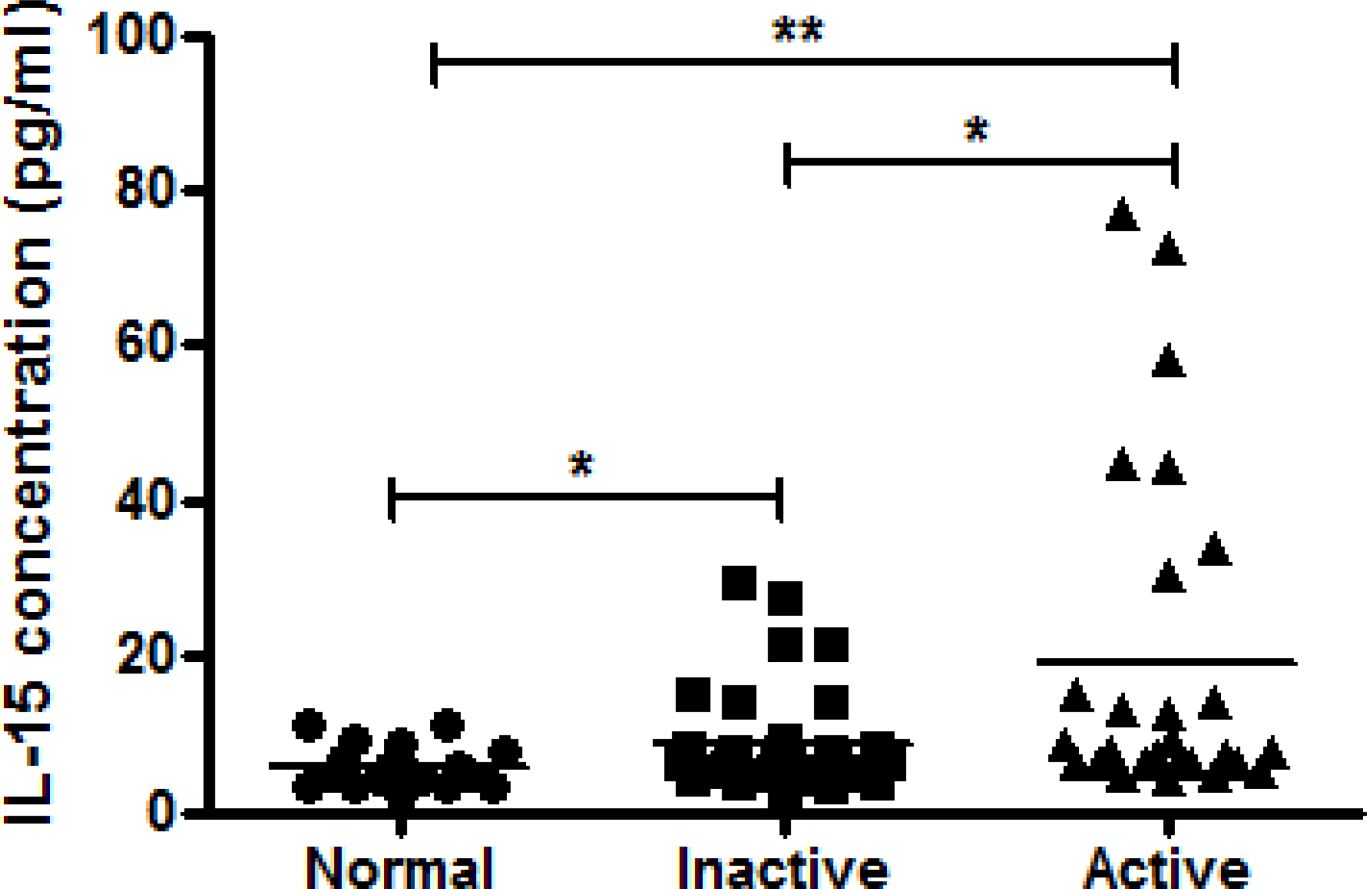
Comparison of serum levels of IL-15 determined by ELISA in samples from healthy controls (*n* = 18) and inactive (*n* = 41) and active (*n* = 27) SLE patients using scattergram (transverse lines indicate means). * p<0.05/**p<0.01, by Mann–Whitney U-test. (calculated by SPSS 17.0 software).

An increased level of IL-15 was detected in sera from active and inactive patients compared with healthy controls (Fig 1). Although no significant correlation was found between disease activity and IL-15 serum levels (r = 0.004, P = 0.649). SLE patients with active disease show higher IL-15 serum levels compared to inactive patients (19.4±4.1 pg/ml vs. 8.6±1.0 pg/ml, p = 0.019)

### Effect of IL-15 on NK cytotoxicity against K562 cells

**Fig 2:**
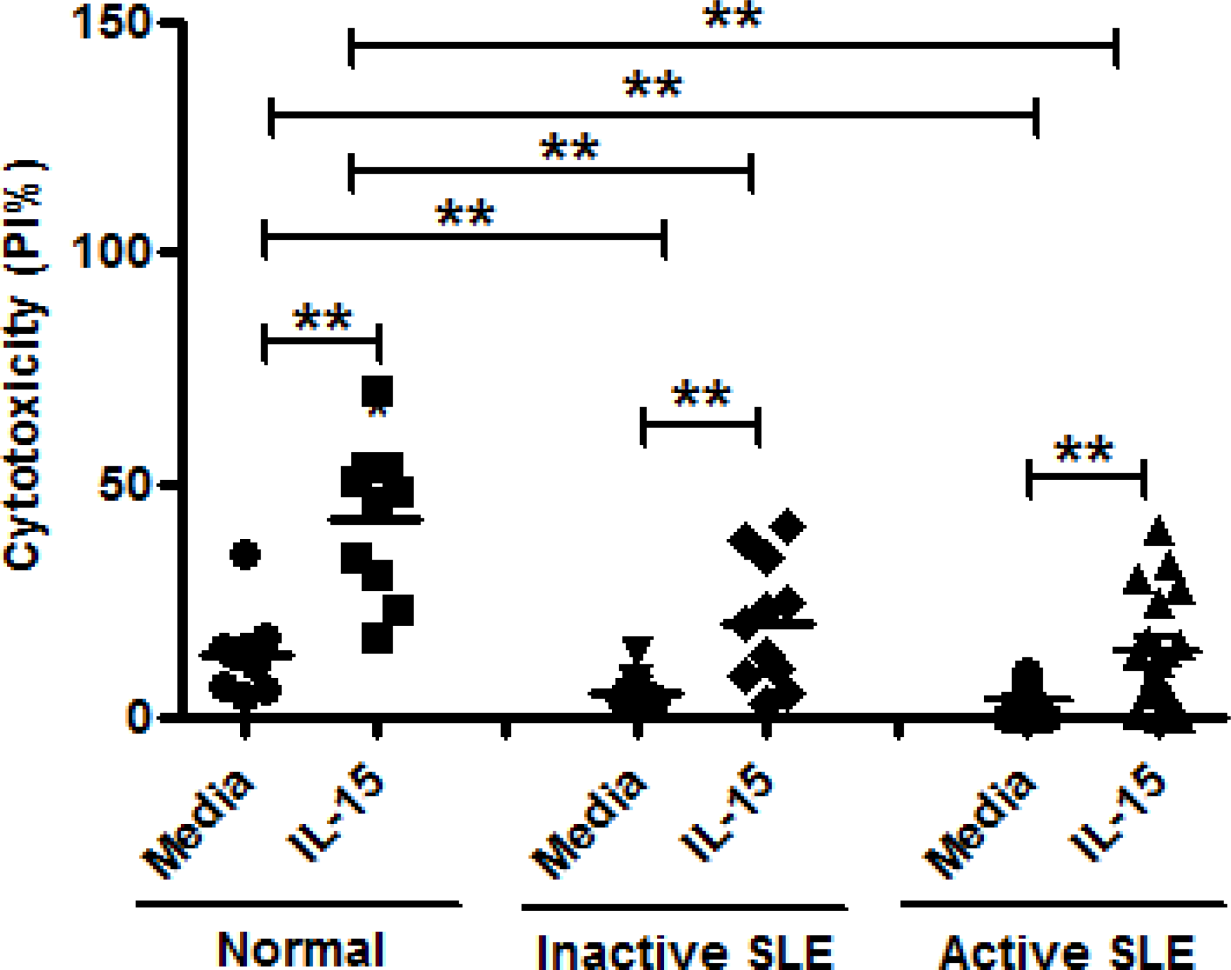
Effect of interleukin (IL)-15 on NK cytotoxicity of mononuclear cells from inactive SLE patients (n = 11), active SLE patients (n = 18) and healthy controls (n = 10). Cytotoxicity was measured by percentage of dead K562 cells expressing PI using effector: target(E:T) ratio of 25:1 **p<0.01, The Wilcoxon signed rank test was applied for analysis of the difference of responses before and after IL-15 treatment. SLE patient and healthy control data were compared between groups using the Mann–Whitney U-test. (calculated by SPSS 17.0 software).

Fig 2 shows the NK cytotoxicity of MNCs from SLE and healthy controls against K562 cells under the influence of IL-15. NK cytotoxicity of SLE patients with inactive disease and SLE patients with active disease was deficient compared to controls at E:T = 25:1 (inactive SLE, 5.1±1.2% vs. 13.6±2.7%, p = 0.005, and active SLE 3.8±0.8% vs. 13.6±2.7%, p = <0.001). IL-15 increased NK cytotoxicity of active SLE patients and inactive SLE, but to a lesser extent compared to IL-15-treated controls (inactive SLE, 20.1±4.0% vs. 5.1±1.2%, p = 0.003, and active SLE 13.8±2.9% vs. 3.8±0.8%, p<0.001). NK cell cytotoxicity in patients with active SLE was not different from those in patients with inactive SLE (P = 0.416)

### Effect of IL-15 on CD69 expression on NK cells

**Fig 3:**
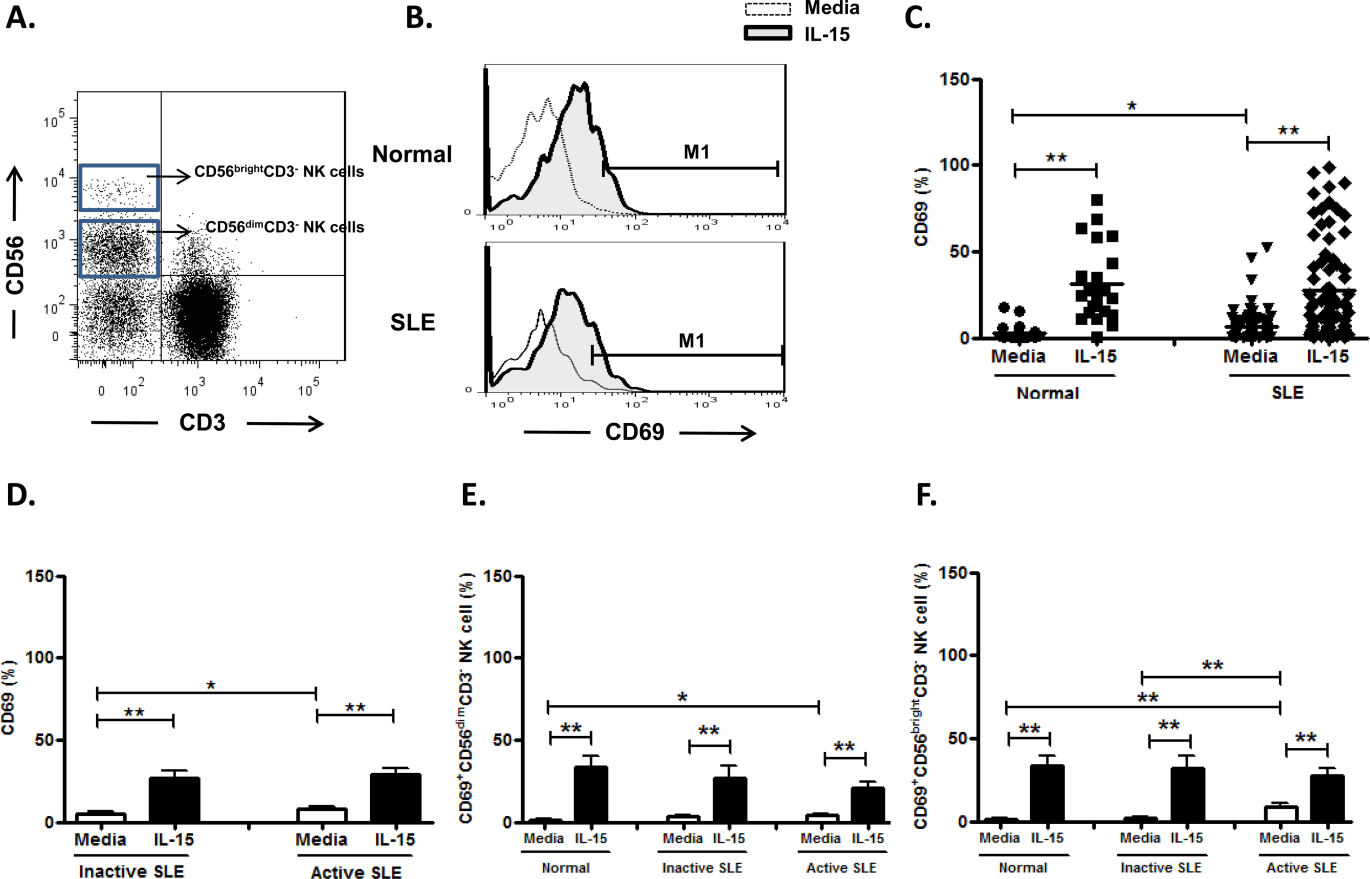
Effect of interleukin (IL)-15 on CD69 expression of CD56^+^CD3^-^ NK cells from SLE patients and healthy controls. A. Gating of CD56^birght^ and CD56^dim^ CD3^-^ NK cells by flow cytometry. B representative profile of Representative histograms of SLE patients (SLE) and controls (Normal); C. Comparison between SLE patients as a whole and normal controls (Normal) using scattergram (transverse lines indicate means); D. Comparison between SLE patients with active disease and inactive disease; E. Comparison of CD69 expression on CD56^dim^ NK subsets in SLE patients with different severity as well as normal controls; F. Comparison of CD69 expression on CD56^bright^ NK subsets in SLE patients with different severity as well as normal controls. PBMC were stimulated with or without IL-15 (10ng/ml) for 18hrs For D,E,F, data was expressed as mean percent expression (%) ± S.E.M. (Normal, n = 25; SLE, total n = 84, SLE with active disease, n = 44, SLE with inactive disease, n = 40) * p<0.05, ** p<0.01, The Wilcoxon signed rank test was applied for analysis of the difference of responses before and after IL-15 treatment. SLE patient and healthy control data were compared between groups using the Mann–Whitney U-test. (calculated by SPSS 17.0 software).

NK cells from SLE patients expressed higher CD69 compared to controls (6.5±1.0%vs. 3.0±0.9%, p = 0.031). (Fig 3B and Fig 3C) We observed a higher frequency of CD69 expressing NK cells in active disease compared to those with inactive disease (7.8±1.6% vs. 5.0±1.1%, p = 0.049) (Fig 3D). IL-15 enhanced CD69 expression on NK cells of patients with inactive disease (26.5±4.7% vs. 5.0±1.1%, p<0.001), as well as patients in active SLE disease (28.7±4.4% vs. 7.8±1.6%, p<0.001). SLE with active disease had higher CD69 expression on CD56^bright^ NK cells compared to those with inactive disease (8.7±2.4% vs. 2.2±0.8, p = 0.004). However, their response to IL-15 did not differ (Fig 3E and 3F).

### Effect of IL-15 on CD94 expression on NK cells

**Fig 4:**
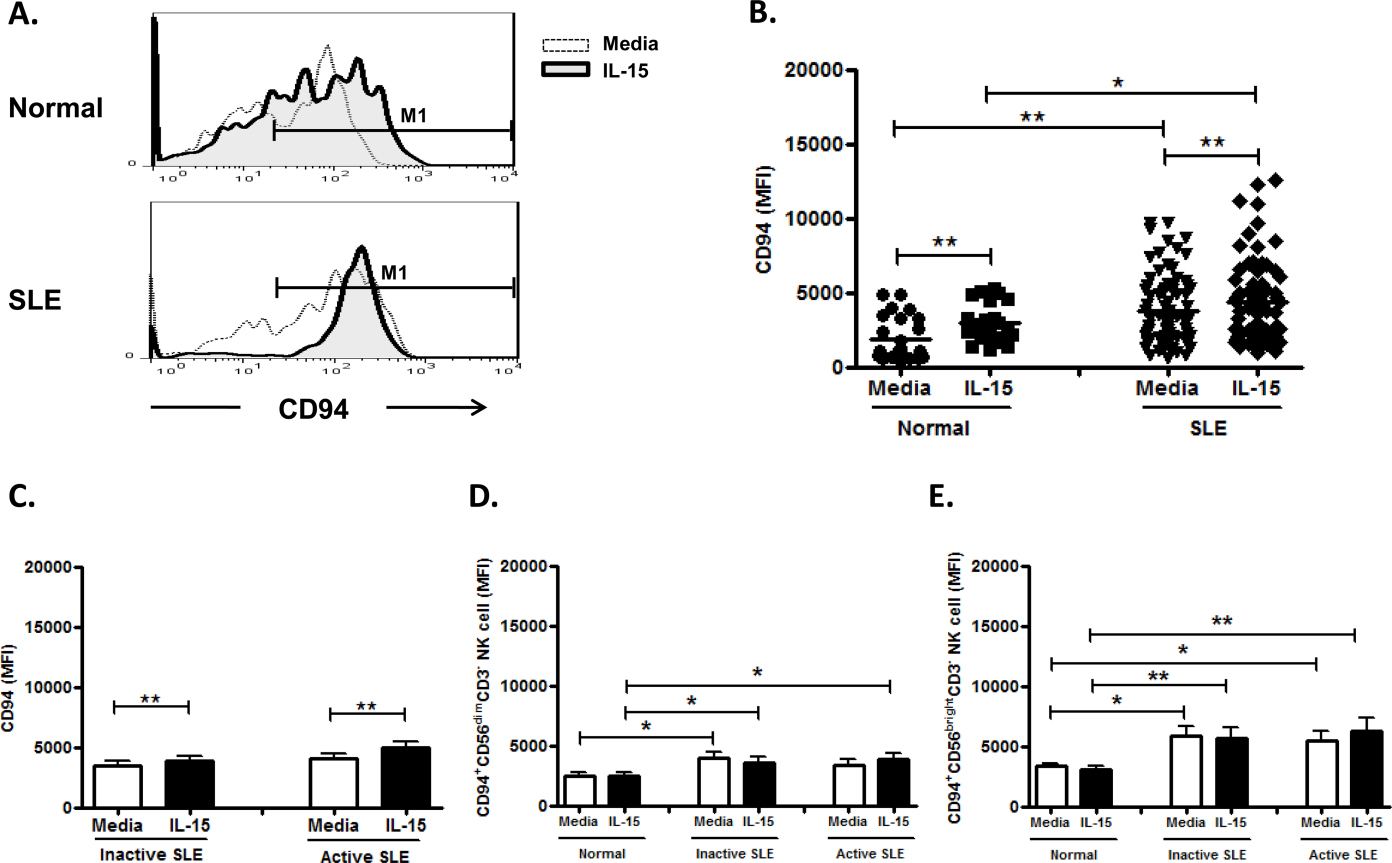
Effect of interleukin (IL)-15 on CD94 expression of CD56^+^CD3^-^ NK cells from SLE patients and healthy controls. A. Representative histograms of SLE patients (SLE) and controls (Normal) B.Comparison between SLE patients as a whole and normal controls (Normal) using scattergrams (transverse lines indicate means) C. Comparison between SLE patients with active disease and inactive disease; D. Comparison of CD94 expression on CD56^dim^ NK subsets in SLE patients with different severity as well as normal controls; E. Comparison of CD94 expression on CD56^bright^ NK subsets in SLE patients with different severity as well as normal controls. PBMC were stimulated with or without IL-15 (10ng/ml) for 18hrs. For C,D,E, data was expressed as mean percent expression (%) ± S.E.M. (Normal, n = 25; SLE, total n = 80, SLE with active disease, n = 40, SLE with inactive disease, n = 40) * p<0.05, ** p<0.01, The Wilcoxon signed rank test was applied for analysis of the difference of responses before and after IL-15 treatment. SLE patient and healthy control data were compared between groups using the Mann–Whitney U-test. (calculated by SPSS 17.0 software).

As nearly all NK cells expressed CD94, we use the mean fluorescence intensity (MFI) to measure CD94 expression. As shown in Fig 4A and Fig 4B, the CD94 expression on NK cells from SLE disease was higher than controls (3808.6±258.5 vs. 1860.8±304.5, p<0.001). However, there was no significant difference in CD94 MFI between SLE with active and inactive disease (4110.1±386.3 vs. 3507.1±341.8, p = 0.224) (Fig 4C). IL-15 enhanced CD94 expression on NK cells from controls (2949.9±269.7 vs. 1860.8±304.5, p = 0.001), inactive SLE disease (3882.0±413.6 vs. 3507.1±341.8, p = 0.003), and active SLE disease (4921.1±513.3 vs. 4110.1±386.3, p = 0.001), respectively. CD94 expression was higher in CD56^bright^ NK cells than in CD56^dim^ NK cells (controls, 3341.3±244.4 vs. 2505.2±235.7, p = 0.005; inactive SLE, 5866.0±764.4 vs. 4006.5±463.2, p = 0.099; cctive SLE, 5481.3±795.2 vs. 3327.9±499.4, p = 0.033) (Fig 4D and Fig 4E).

### Effect of IL-15 on NKG2A expression on NK cells

**Fig 5:**
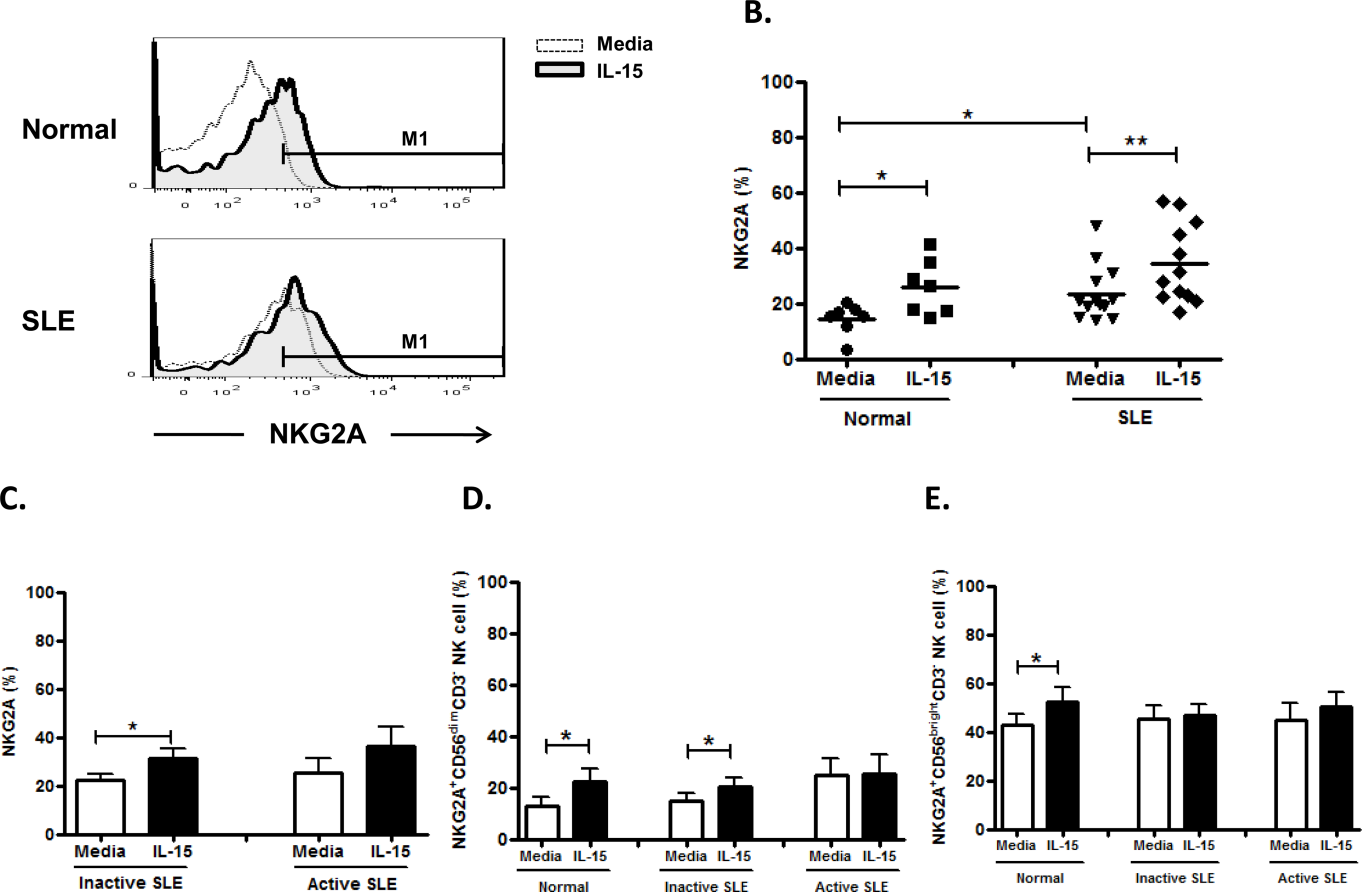
Effect of interleukin(IL)-15 on NKG2A expression of CD56^+^CD3^-^ NK cells from SLE patients and healthy controls. A. Representative histograms of SLE patients (SLE) and controls (Normal) B.Comparison between SLE patients as a whole and normal controls (Normal) using scattergrams (transverse lines indicate means); C. Comparison between SLE patients with active disease and inactive disease; D. Comparison of NKG2A expression on CD56^dim^ NK subsets in SLE patients with different severity as well as normal controls; E. Comparison of NKG2A expression on CD56^bright^ NK subsets in SLE patients with different severity as well as normal controls. PBMC were stimulated with or without IL-15 (10ng/ml) for 18hrs. For C,D,E, data was expressed as mean percent expression (%) ± S.E.M. (Normal, n = 7; SLE, total n = 13, SLE with active disease, n = 5, SLE with inactive disease, n = 8) * p<0.05, ** p<0.01, The Wilcoxon signed rank test was applied for analysis of the difference of responses before and after IL-15 treatment. SLE patient and healthy control data were compared between groups using the Mann–Whitney U-test. (calculated by SPSS 17.0 software).

As shown in Fig 5A and Fig 5B, the NKG2A expression on NK cells from SLE patients was higher than controls. (23.5±2.7% vs. 14.5±2.1%, p = 0.029). However, there was no significant difference in the NKG2A expression between SLE patients with active and inactive disease (25.5±6.0% vs. 22.2±2.7%, p = 0.942) (Fig 5C). IL-15 enhanced NKG2A expression on NK cells from controls (25.8±3.7% vs. 14.5±2.1%, p = 0.028), inactive SLE disease (31.2±4.1% vs. 22.2±2.7%, p = 0.025), but not active SLE disease (36.1±8.3% vs. 25.5±6.0%, p = 0.225). NKG2A expression was higher in CD56^bright^ NK cells than in CD56^dim^ NK cells (controls, 42.7±4.6% vs. 12.9±3.5%, p = 0.001; inactive SLE, 45.1±5.6% vs. 14.9±2.8%, p = 0.001; active SLE, 45.0±6.9% vs. 24.6±6.5%, p = 0.048) (Fig 5D and Fig 5E).

### Effect of IL-15 on NKp30 expression on NK cells

**Fig 6:**
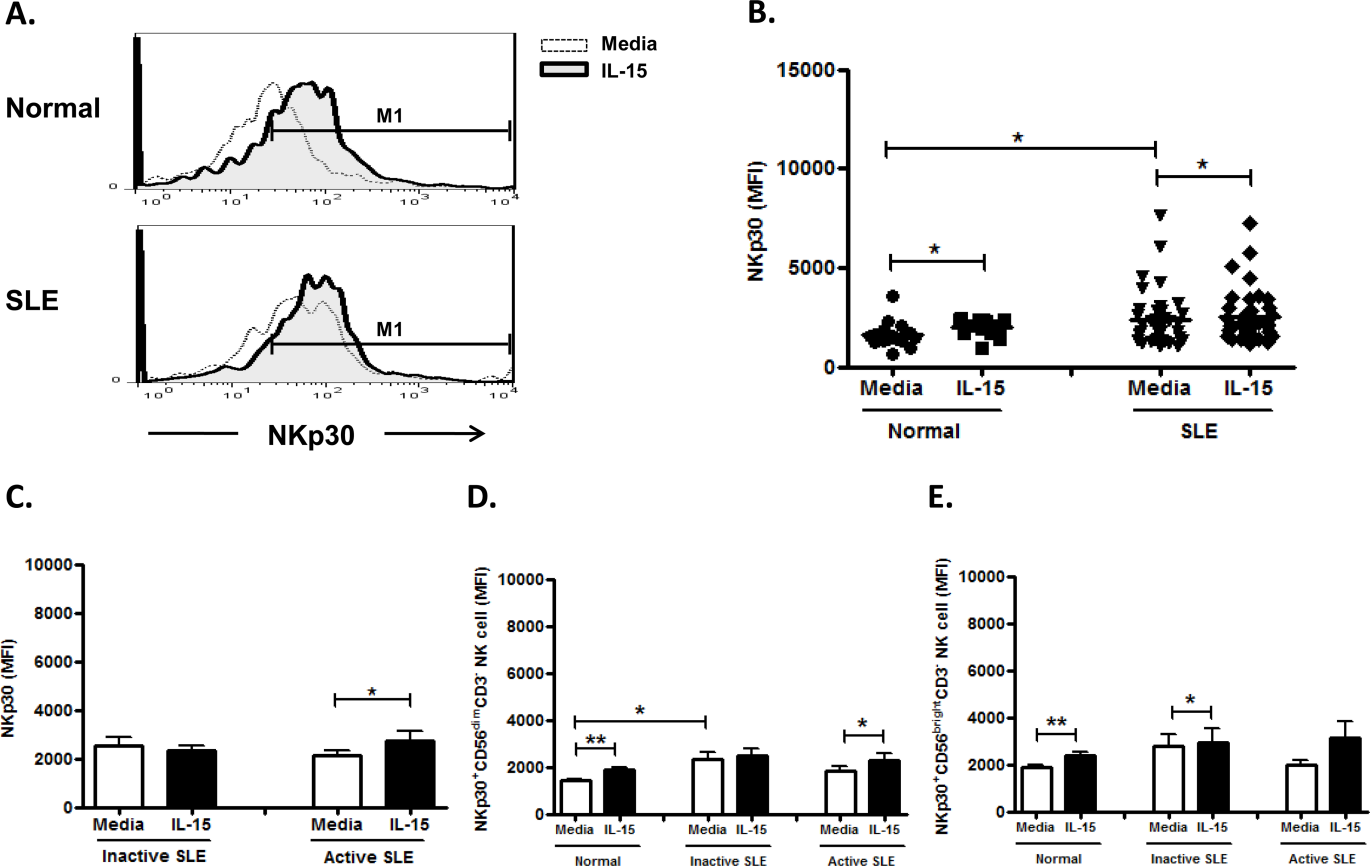
Effect of interleukin(IL)-15 on NKp30 expression of CD56^+^CD3^-^ NK cells from SLE patients and healthy control. A. Representative histograms of SLE patients (SLE) and controls(Normal); B. Comparison between SLE patients as a whole and controls (Normal) using scattergrams (transverse lines indicate means); C. Comparison between SLE patients with active disease and inactive disease; D. Comparison of NKp30 expression on CD56^dim^ NK subsets in SLE patients with different severity as well as normal controls; E. Comparison of NKp30 expression on CD56^bright^ NK subsets in SLE patients with different severity as well as normal controls. PBMC were stimulated with or without IL-15 (10ng/ml) for 18hrs For C,D,E, data was expressed as mean percent expression (%) ± S.E.M. (Normal, n = 16; SLE, total n = 41, SLE with active disease, n = 18, SLE with inactive disease, n = 23) * p<0.05, ** p<0.01, The Wilcoxon signed rank test was applied for analysis of the difference of responses before and after IL-15 treatment. SLE patient and healthy control data were compared between groups using the Mann–Whitney U-test. (calculated by SPSS 17.0 software).

As the majority of NK cells expressed NKp30, we use the mean fluorescence intensity (MFI) to measure the level of marker expression. NKp30 expression on NK cells from SLE patient was higher than controls (2358.5±226.0 vs. 1618.3±163.1, p = 0.041) (Fig 6A and Fig 6B), but seemed unrelated to disease activity (Fig 6C). IL-15 enhance the NKp30 expression on control NK cells (2009.1±104.8 MFI vs. 1618.3±163.1 MFI, p = 0.030) and NK cell from active SLE disease (2734.8±379.2 MFI vs. 2151.0±202.4 MFI, p = 0.030), but failed to enhance that NK cells from inactive SLE diseases (2339.9±211.2 vs. 2509.5±362.8, p = 0.212). There was no significant difference with regards to NKp30 expression between CD56 ^bright^ NK cells and CD56^dim^ subsets. (Fig 6D and Fig 6E)

### Effect of IL-15 on NKp46 expression on NK cells

**Fig 7:**
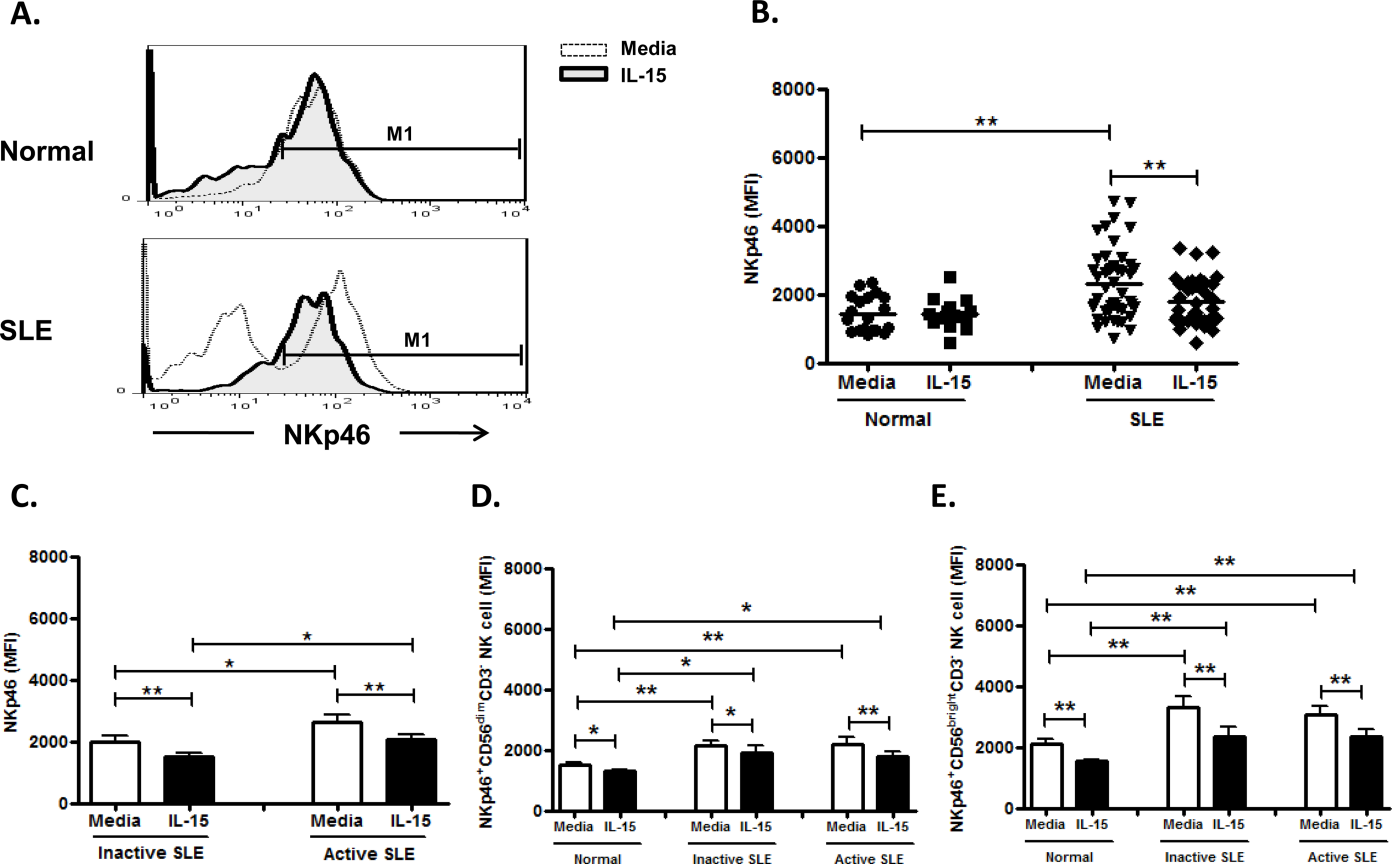
Effect of interleukin (IL)-15 on NKp46 expression of CD56^+^CD3^-^ NK cells from SLE patients and healthy control. A. Representative histograms of SLE patients (SLE) and controls(Normal); B. Comparison between SLE patients as a whole and controls (Normal) using scattergrams (transverse lines indicate means); C. Comparison between SLE patients with active disease and inactive disease; D. Comparison of NKp46 expression on CD56^dim^ NK subsets in SLE patients with different severity as well as normal controls; E. Comparison of NKp46 expression on CD56^bright^ NK subsets in SLE patients with different severity as well as normal controls. PBMC were stimulated with or without IL-15 (10ng/ml) for 18hrs For C,D,E, data was expressed as mean percent expression (%) ± S.E.M. (Normal, n = 19; SLE, total n = 47, SLE with active disease, n = 23, SLE with inactive disease, n = 24) * p<0.05, ** p<0.01, The Wilcoxon signed rank test was applied for analysis of the difference of responses before and after IL-15 treatment. SLE patient and healthy control data were compared between groups using the Mann–Whitney U-test. (calculated by SPSS 17.0 software).

As shown in Fig 7A, In contrast to single peak expression of NKp46 on control NK cells, the majority of SLE patients (n = 46) had a bimodal distribution of NKp46 expression on their NK cells, namely, NKp46^+^ and NKp46^-^ subsets. Only 9 patients with inactive SLE disease showed single peak NKp46 expression as observed in controls.

Using MFI gating on NKp46^+^ population, NK cells from SLE patients expressed higher NKp46 compared to healthy controls (2309.0±149.7 vs. 1440.0±119.4, p = 0.001) (Fig 7B). The SLE patients with active disease have higher NKp46 expression on their NK cells compared to those with inactive disease (2639.3±212.6 MFI vs. 2006.3±194.8 MFI, p = 0.045) (Fig 7C). IL-15, in contrast to NKp30, down-regulate the NKp46 expression in SLE patients (1785.3±105.1 MFI vs. 2309.0±149.7 MFI, p<0.001) as well as controls (1426.8±106.9 MFI vs. 1440.0±119.4 MFI, p = 0.717). As shown in Fig 7D and 7E, NKp46 expression were much higher on CD56 ^bright^ NK subsets, compared to that on CD56^dim^ subsets, in controls and SLE patient s alike (controls: 2124.1±142.8 vs. 1517.3±65.0, p = 0.001; inactive SLE: 3290.9±362.7 vs. 2154.1±170.9, p = 0.024; active SLE: 3058.0±270.9 vs. 2190.4±217.2, p = 0.039).

### Effect of IL-15 on NKG2D expression on NK cells

**Fig 8:**
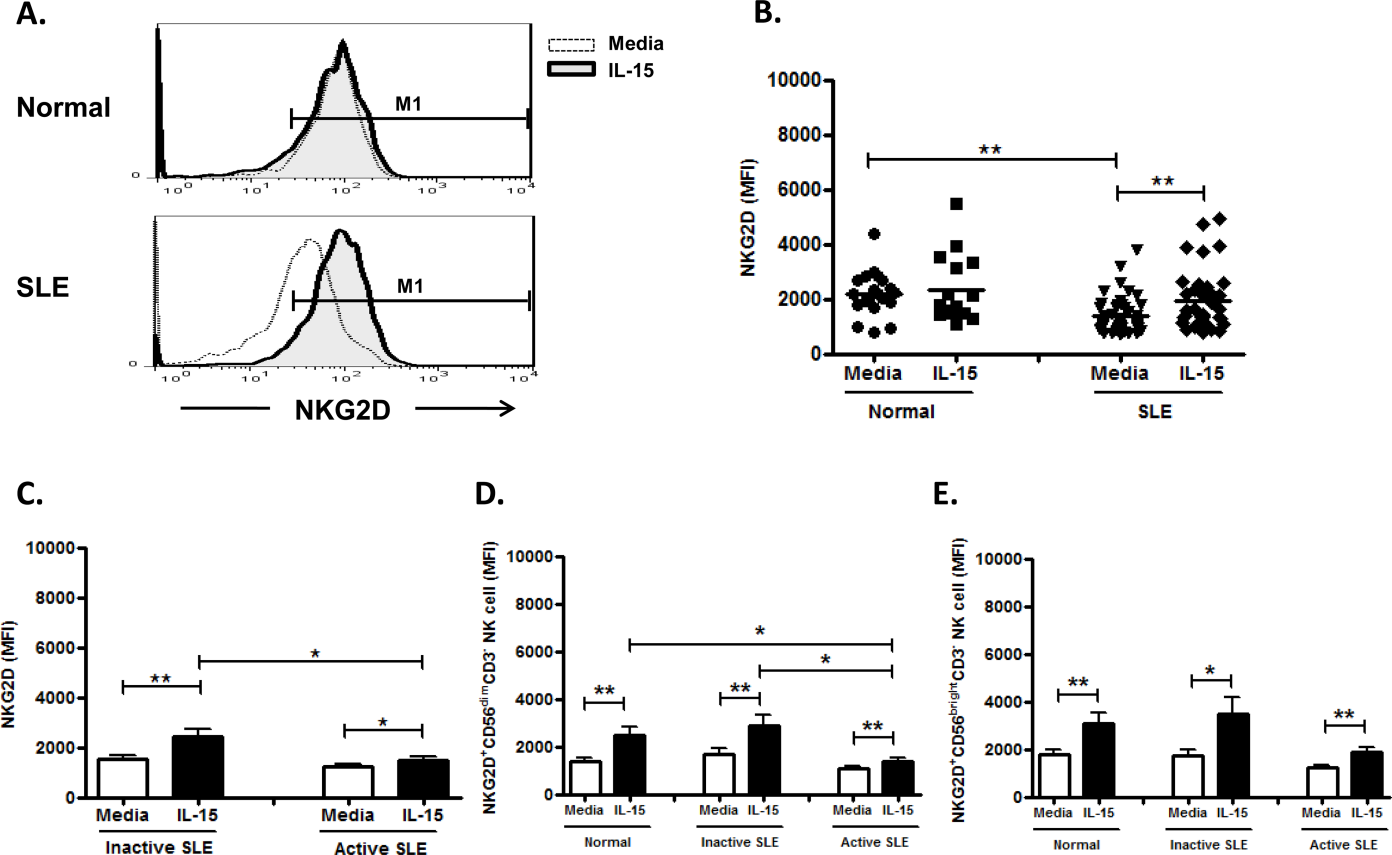
Effect of interleukin (IL)-15 on NKG2D expression of CD56^+^CD3^-^ NK cells from SLE patients and healthy control. A. Representative histograms of SLE patients (SLE) and controls(Normal);B Comparison between SLE patients as a whole and controls (Normal) using scattergrams (transverse lines indicate means); C. Comparison between SLE patients with active disease and inactive disease; D. Comparison of NKG2D expression on CD56^dim^ NK subsets in SLE patients with different severity as well as normal controls; E. Comparison of NKG2D expression on CD56^bright^ NK subsets in SLE patients with different severity as well as normal controls. PBMC were stimulated with or without IL-15 (10ng/ml) for 18hrs For C,D,E, data was expressed as mean percent expression (%) ± S.E.M. (Normal, n = 20; SLE, total n = 46, SLE with active disease, n = 25, SLE with inactive disease, n = 21) * p<0.05, ** p<0.01. The Wilcoxon signed rank test was applied for analysis of the difference of responses before and after IL-15 treatment. SLE patient and healthy control data were compared between groups using the Mann–Whitney U-test. (calculated by SPSS 17.0 software).

NK cells from SLE patients expressed decreased NKG2D compared to healthy controls (1361.8±98.1 vs. 2192.0±180.5, p<0.001) (Fig 8A and Fig 7B). No difference of NKG2D expression on NK cells were found between active and inactive SLE diseases (1237.7±111.1 vs. 1509.5±166.7 MFI, p = 0.275) (Fig 8C). IL-15 enhanced the NKG2D expression of NK cell in SLE patients (1921.9±178.9 MFI vs. 1361.8±98.1 MFI, p<0.001), but not controls (2317.1±305.4 MFI vs. 2192.0±180.5 MFI, p = 0.959). As shown in Fig 8D and Fig 8E, NKG2D were comparably expressed on CD56 ^bright^ and CD56^dim^ populations.

### Effect of IL-15 on NKAT-2, CD158a, CD158b, CD158k expression on NK cells

**Fig 9:**
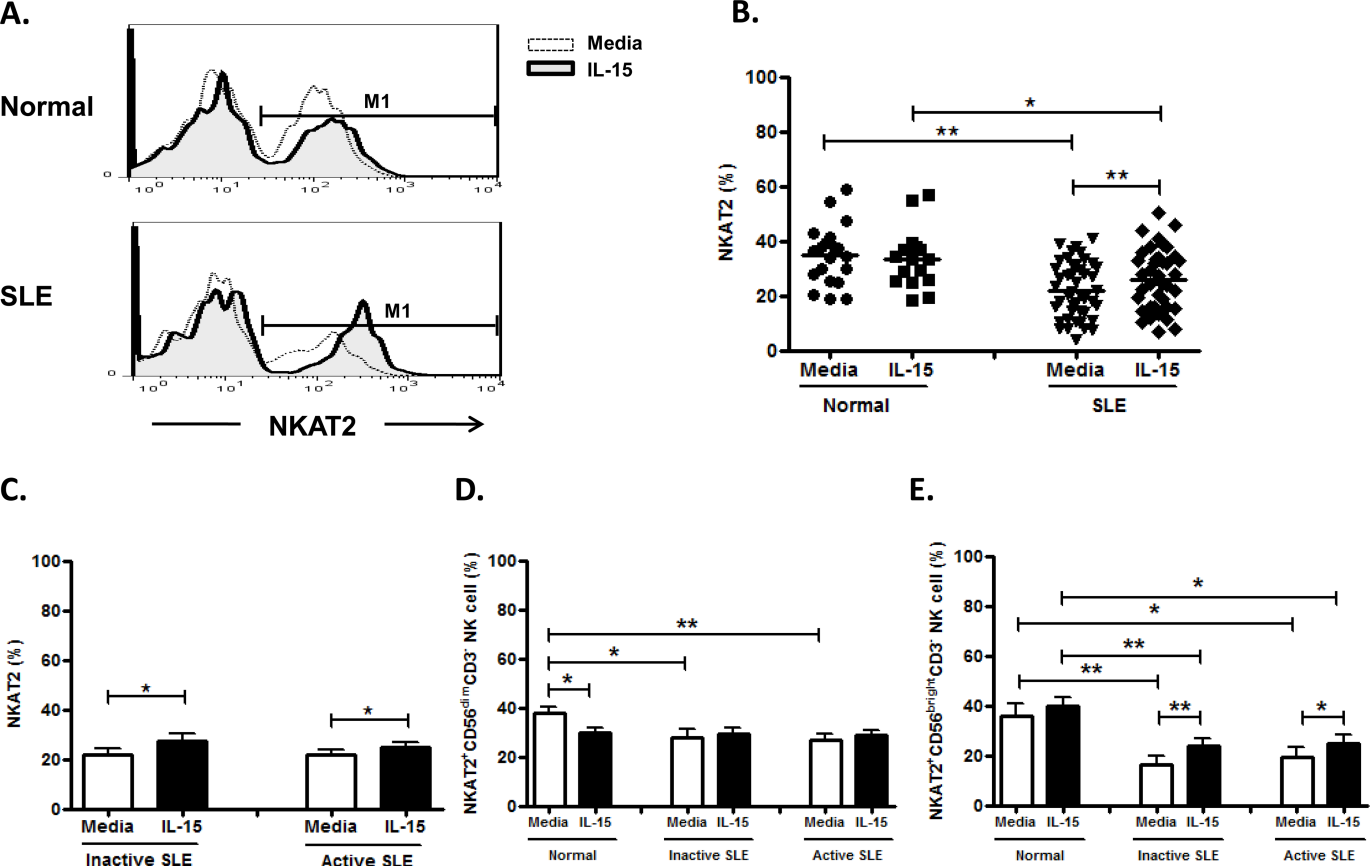
Effect of interleukin(IL)-15 on NKAT2 expression of CD56^+^CD3^-^ NK cells from SLE and healthy control. A. Representative histograms of SLE patients (SLE) and controls(Normal) B.Comparison between SLE patients as a whole and controls (Normal) using scattergrams (transverse lines indicate means); C. Comparison between SLE patients with active disease and inactive disease; D. Comparison of NKAT2 expression on CD56^dim^ NK subsets in SLE patients with different severity as well as normal controls; E. Comparison of NKAT2 expression on CD56^bright^ NK subsets in SLE patients with different severity as well as normal controls. PBMC were stimulated with or without IL-15 (10ng/ml) for 18hrs. For C,D,E, data was expressed as mean percent expression (%) ± S.E.M. (Normal, n = 19; SLE, total n = 46, SLE with active disease, n = 27, SLE with inactive disease, n = 18) * p<0.05, ** p<0.01. The Wilcoxon signed rank test was applied for analysis of the difference of responses before and after IL-15 treatment. SLE patient and healthy control data were compared between groups using the Mann–Whitney U-test. (calculated by SPSS 17.0 software).

As shown in Fig 9A, NK cells can be separated into NKAT-2^+^ and NKAT-2^-^ population in both SLE and controls. Overall speaking, NK cells from SLE patients expressed decreased NKAT-2 compared to healthy controls (22.0±1.5% vs. 34.1±2.5%, p<0.001). IL-15 enhanced NKAT-2 expression on NK cells from SLE patients (25.8±1.8% vs. 22.0±1.5%, p = 0.001) but had no effect on NKAT-2 expression on control NK cells (33.5±2.7% vs. 34.1±2.5%, p = 0.155) (Fig 9A and 9B). NKAT-2 did not differ between active and inactive diseases of SLE (22.0±1.8% vs. 22.0±2.6%, p = 0.982) (Fig 9C). NKAT-2 on CD56^bright^ NK subsets seemed to be more responsive to IL-15 stimulation compared to CD56^dim^ NK subsets.(Fig 9D and Fig 9E)

**Fig 10:**
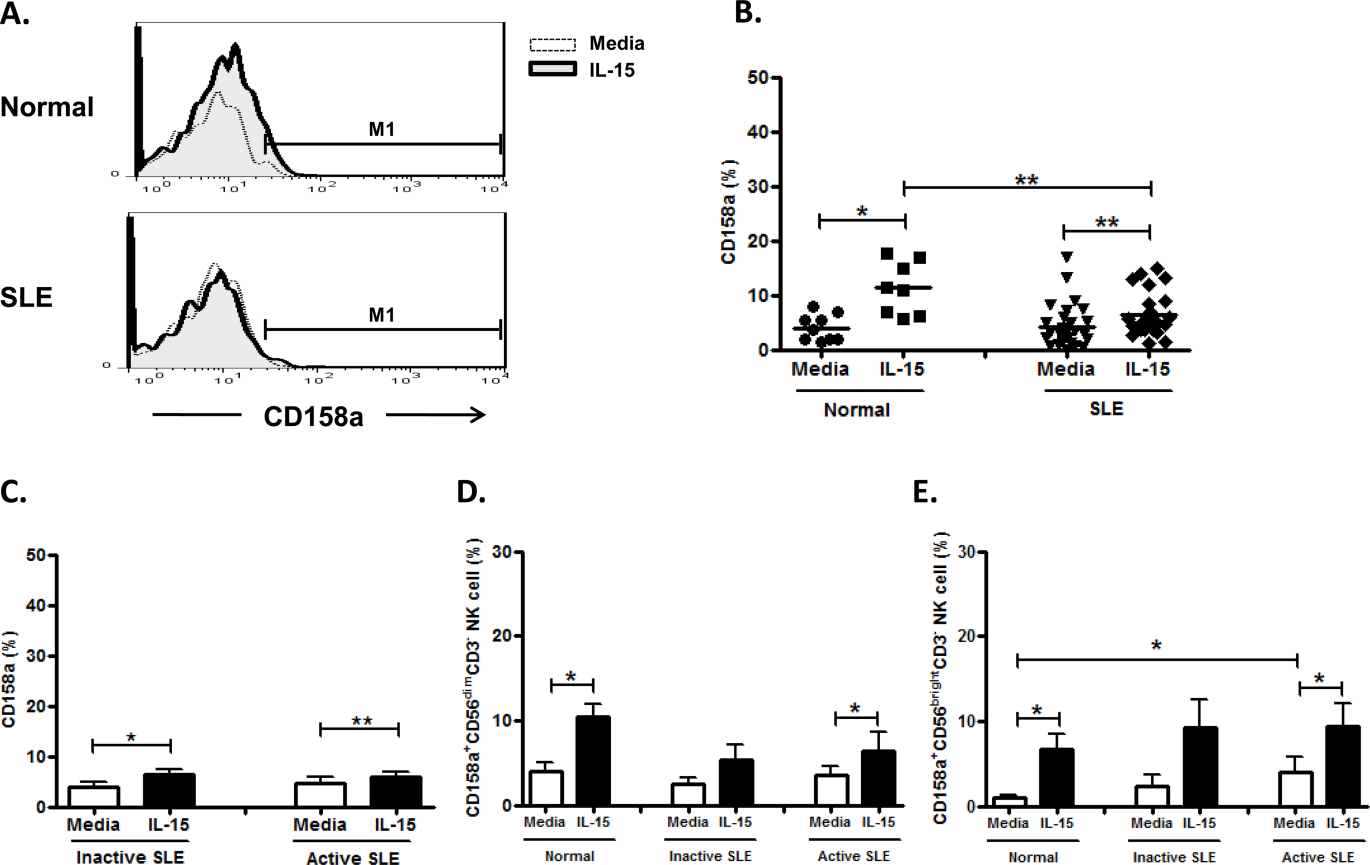
Effect of interleukin(IL)-15 on CD158a expression of CD56^+^CD3^-^ NK cells from SLE patients and healthy control. A. Representative histograms of SLE patients (SLE) and controls(Normal);B Comparison between SLE patients as a whole and controls (Normal) using scattergrams (transverse lines indicate means); C. Comparison between SLE patients with active disease and inactive disease; D. Comparison of CD158a expression on CD56^dim^ NK subsets in SLE patients with different severity as well as normal controls; E. Comparison of CD158a expression on CD56^bright^ NK subsets in SLE patients with different severity as well as normal controls. PBMC were stimulated with or without IL-15 (10ng/ml) for 18hrs. For C,D,E, data was expressed as mean percent expression (%) ± S.E.M. (Normal, n = 9; SLE, total n = 28, SLE with active disease, n = 11, SLE with inactive disease, n = 17) * p<0.05, ** p<0.01.The Wilcoxon signed rank test was applied for analysis of the difference of responses before and after IL-15 treatment. SLE patient and healthy control data were compared between groups using the Mann–Whitney U-test. (calculated by SPSS 17.0 software).

CD158a expression was comparable in SLE patient and controls (4.3±0.7% vs. 4.0±1.0%, p = 0.804) (Fig 10A and Fig 10B). IL-15 enhanced CD158a expression in SLE patient (6.5±0.8% vs. 4.3±0.7%, p<0.001) as well as controls (11.3±1.7% vs. 4.0±0.8%, p = 0.012). However, CD158a did not differ between active and inactive diseases of SLE (4.6±1.2% vs. 3.7±1.0%, p = 0.352) (Fig 10C). Comparable expression of CD158a was expressed on CD56^dim^ and CD56^bright^ NK subsets. (Fig 10D and Fig 10E)

**Fig 11:**
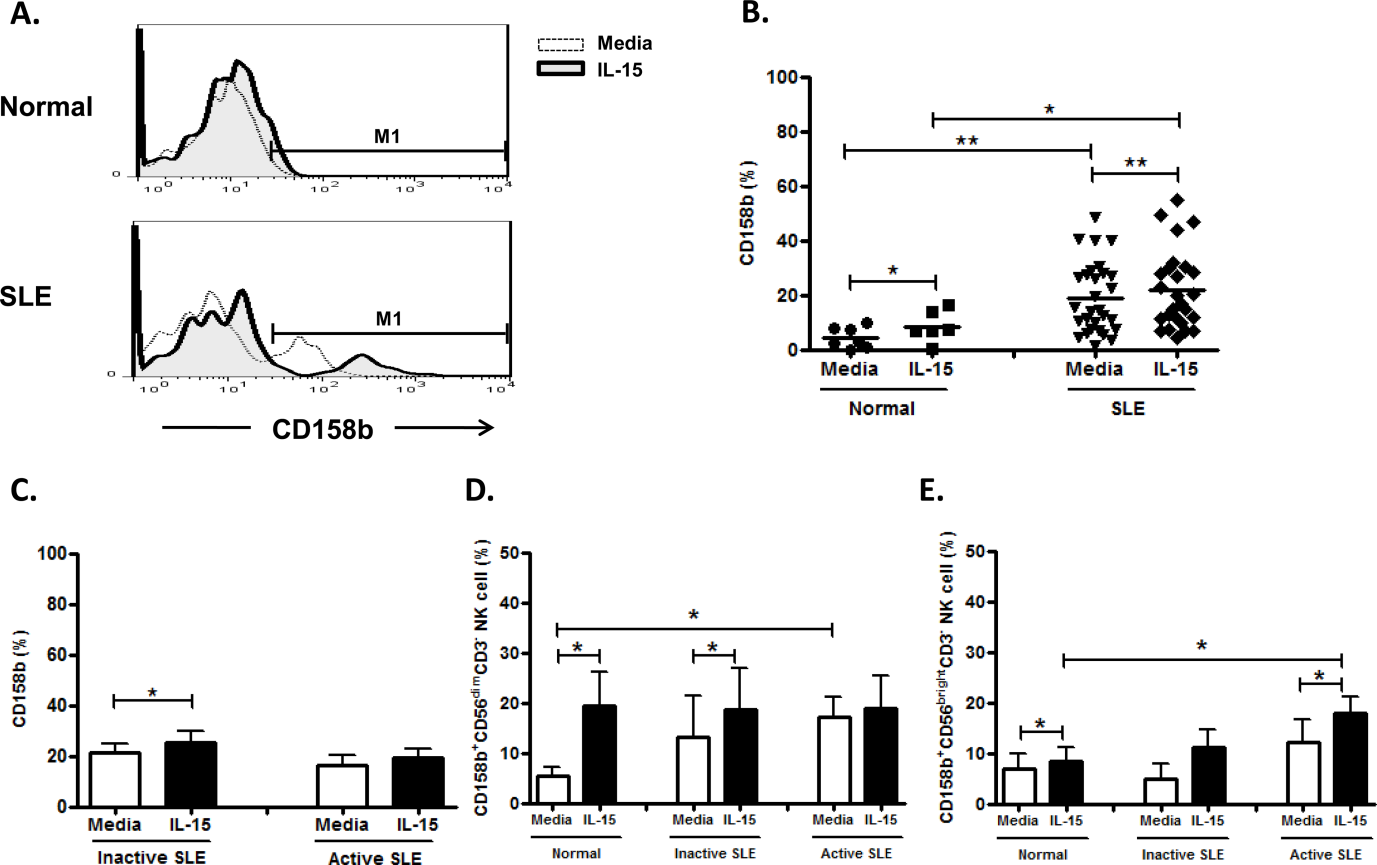
Effect of interleukin(IL)-15 on CD158b expression of CD56^+^CD3^-^ NK cells from SLE patients and healthy control. A. Representative histograms of SLE patients (SLE) and controls(Normal); B. Comparison between SLE patients as a whole and controls (Normal) using scattergrams (transverse lines indicate means); C. Comparison between SLE patients with active disease and inactive disease; D. Comparison of CD158b expression on CD56^dim^ NK subsets in SLE patients with different severity as well as normal controls; E. Comparison of CD158b expression on CD56^bright^ NK subsets in SLE patients with different severity as well as normal controls. PBMC were stimulated with or without IL-15 (10ng/ml) for 18hrs For C,D,E, data was expressed as mean percent expression (%) ± S.E.M. (Normal, n = 7; SLE, total n = 28, SLE with active disease, n = 13, SLE with inactive disease, n = 15) * p<0.05, ** p<0.01, The Wilcoxon signed rank test was applied for analysis of the difference of responses before and after IL-15 treatment. SLE patient and healthy control data were compared between groups using the Mann–Whitney U-test. (calculated by SPSS 17.0 software).

Distinct from controls, NK cells from SLE can be separated into CD158b^+^ and CD158b^-^ subsets (Fig 11A). NK cells from SLE patients expressed increased CD158b compared to controls (19.2±2.4% vs. 4.4±1.6%, p = 0.002). (Fig 11A and Fig 11B) CD158b on NK cells from SLE and controls was enhanced with IL-15 (SLE, 22.5±2.9% vs. 19.2±2.4%, p = 0.003; Controls, 8.5±2.4% vs. 4.4±1.5%, p = 0.028) (Fig 11A). However, CD158b expression was unrelated to SLE disease activity (Fig 11C). CD158b expression was much higher on CD56^dim^ NK subsets, compared to that on CD56^brigh^ subsets in SLE patients (Fig 11D and Fig 11E).

**Fig 12:**
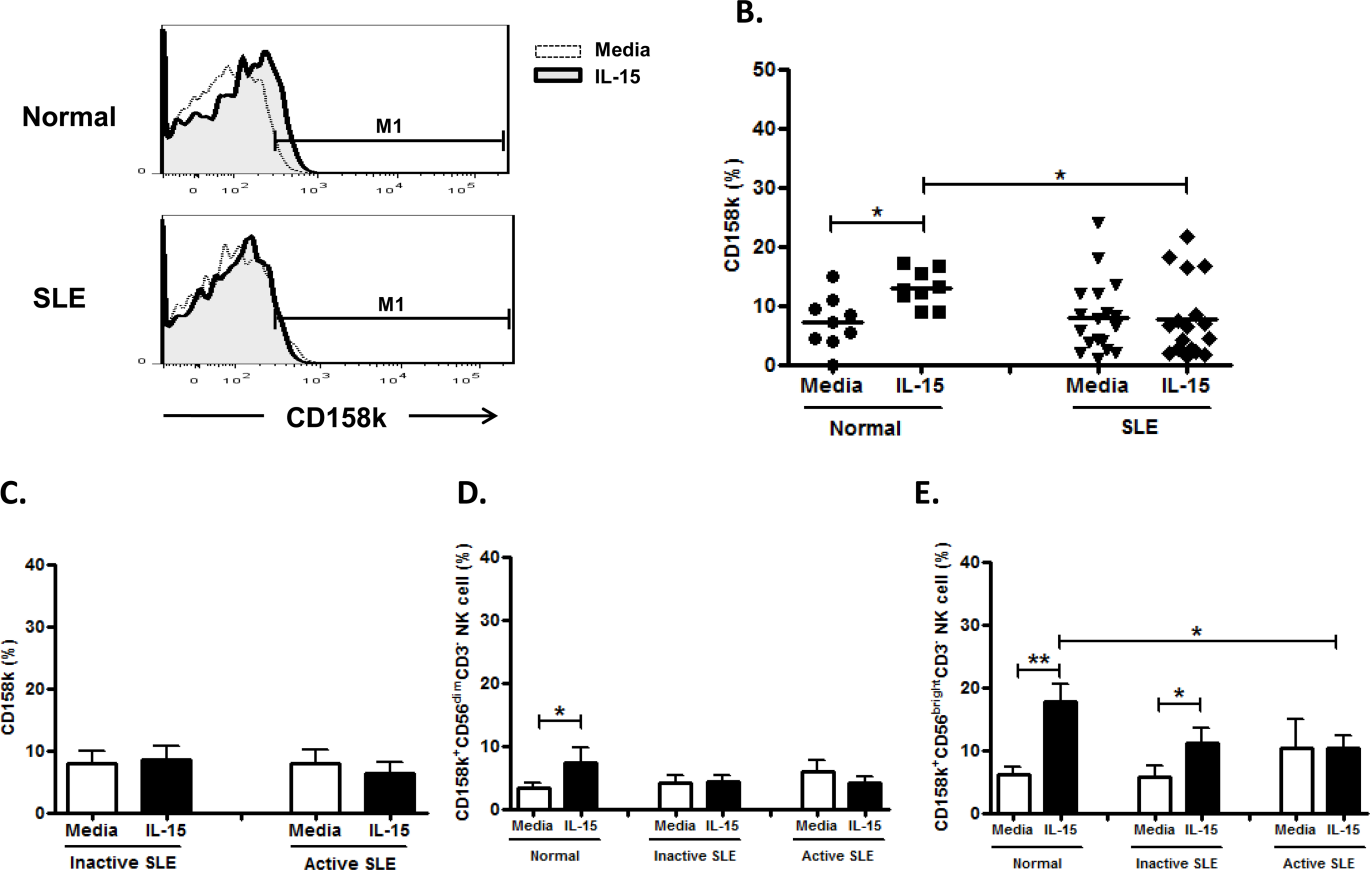
Effect of interleukin(IL)-15 on CD158k expression of CD56^+^CD3^-^ NK cells from SLE patients and healthy control. A. Representative histograms of SLE patients (SLE) and controls(Normal); B. Comparison between SLE patients as a whole and controls (Normal) using scattergrams (transverse lines indicate means); C. Comparison between SLE patients with active disease and inactive disease; D. Comparison of CD158k expression on CD56^dim^ NK subsets in SLE patients with different severity as well as normal controls; E. Comparison of CD158k expression on CD56^bright^ NK subsets in SLE patients with different severity as well as normal controls. PBMC were stimulated with or without IL-15 (10ng/ml) for 18hrs For C,D,E, data was expressed as mean percent expression (%) ± S.E.M. (Normal, n = 9; SLE, total n = 18, SLE with active disease, n = 7, SLE with inactive disease, n = 11)* p<0.05, ** p<0.01, The Wilcoxon signed rank test was applied for analysis of the difference of responses before and after IL-15 treatment. SLE patient and healthy control data were compared between groups using the Mann–Whitney U-test. (calculated by SPSS 17.0 software).

NK cells from SLE patients expressed CD158k comparably to healthy controls (7.9±1.4% vs. 7.2±1.5%, p = 0.979). (Fig 12A and Fig 12B). CD158k on NK cells from healthy controls was enhanced with IL-15 (13.0±1.0% vs. 7.2±1.5%, p = 0.021), but not in SLE patients (7.6±1.6% vs. 7.9±1.4%, p = 0.723). However, CD158k was unrelated to disease activity (Fig 12C). CD158k expression on CD56 ^bright^ NK subsets were higher than that on CD56^dim^ subsets in SLE patients (Fig 12D and Fig 12E).

## Discussion

The role of NK cells in the development of human SLE remained poorly understood. In the present study, we sought to examine the NK cytotoxicity, expression of activating NCR and inhibitory NKR on NK cells from SLE patients and determine their response to exogenous IL-15.

We observed higher serum IL-15 levels in SLE patients compared to controls. Patients with active SLE disease have higher IL-15 levels than those with inactive disease. Our finding is consistent with Zhu et al who recently showed that the serum level of IL-15 was significantly elevated in SLE patients and correlated with their mRNA expression [30]. The upregulation of IL-15 may be regulated by hypomethylated CpG in the promoter region of the gene. Overexpression of IL-15 correlated with the development of murine lupus [31]. Circulating NK cells in SLE may be preactivated by IL-15. However, the percentages of NK cells are decreased in PBMC from SLE patients compared to controls.

We found that NK cytotoxicity of SLE patients was decreased compared to controls, consistent with previous studies.[7,8,10] NK cytotoxicity of SLE patients was enhanced by IL-15 to a lesser degree compared to controls. NK cell cytotoxicity against K562 cells, mediated by NK cells, is a complex and dynamic process requiring several different signals, including adhesion, activation, and degranulation. A IL-15 signaling defect may exist in SLE NK cells.

We found that NK cells, especially the CD56^bright^ subsets, from SLE patients expressed greater CD69, which correlated with disease activity. We found that IL-15 enhanced CD69 expression on NK cells from SLE patients regardless of disease activity, in discrepancy with Baranda et al who reported diminished CD69 response of NK cells to IL-15 [27]. Thus, CD69 expression on NK cells may serve as a disease activity indicator. IL-15 is considered disease-promoting it further enhanced CD69 expression in SLE patients.

CD94 is a C-type lectin required for the dimerization of the CD94/NKG2 family of receptors, which are expressed on NK cells and T cell subsets. We found CD94 expression is increased in SLE NK cells, in contrast to Schepis et al who reported no difference [9]. IL-15 enhanced CD94 expression of NK cells from both SLE and controls, consistent with Bo et al [31]. We found a higher CD94 expression on CD56^bright^ subsets compared to CD56^dim^ NK cells, suggesting their regulatory role. IL-15 may aggravate SLE activity due to its CD94 enhancing effect.

In agreement with previous studies [10,32], we found that NKG2A was over-expressed in NK cells from SLE patients compared to controls. We demonstrated that IL-15 could further enhanced NKG2A expression on SLE NK cells. We also showed that NKG2A expression is higher in CD56^brigth^ NK subsets than CD56^dim^ NK subsets. Again, it seemed that IL-15 is detrimental in SLE patients, as it aggravate the aberrant NKG2A expression.

NKp30 is considered a surrogate marker of NK cell functions in humans [33] that regulates the DC/NK cell cross-talk, and mediates the release of Th1 cytokines [34]. NK cells may promote an NKp30-dependent inflammatory state in salivary glands of patients with primary Sjogren’s syndrome [35]. We observed that NK cells from SLE patient expressed higher NKp30 compared to controls, in contrast to Puxeddu at et al who reported no difference of NK p30 expression between SLE patients and controls [36]. IL-15 enhanced NKp30 expression on NK cells from SLE as well as controls, consistent with previous studies [37,38].

NKp46 activates NK cells by interaction with an, as yet, unknown structure on target cells resulting in increased cytokine production and release of cytolytic granules [39]. NKp46 can also confer tumor cell recognition by NK cells and can specifically bind viral hemagglutinins, suggesting its role in antitumor and antiviral immunity [40, 41]. Interestingly, we found that NK cells from the majority of SLE patient have unique NKp46^-^ subsets not found in controls. (Fig 5A) NK cells from SLE patient expressed higher NKp46 compared to controls, consistent with Schepis etal [9]. Distinct from the response of controls, IL-15 down-regulated NKp46 expression of NK cells from SLE patients, probably due to the prior activation by high serum IL-15 in SLE patients. We did not find any difference of expression of NKp30 and NKp46 between SLE patients with inactive disease and active disease.

Dai et al showed that increased frequencies of NKG2D^+^CD4^+^ T cells are inversely correlated with disease activity in juvenile-onset SLE [42]. We found that NKG2D, another NK-activating receptors in SLE activated by NKG2D ligands, was under-expressed on circulating NK cells from SLE, consistent with previous reports [30,32]. We further found that NKG2D on CD56^dim^ NK cells is lower in SLE with active disease compared to inactive disease. Spada et al reported that NKG2D ligands overexpression in lupus nephritis kidney but not in the periphery [43], suggesting that NKG2D^+^NK cells may migrate to the kidney or other target organs. IL-15 enhanced NKG2D expression of NK cells may facilitate migration of NK cells to target organs in SLE.

Hervier et al showed that KIRs under-expression correlated with disease activity in SLE [10]. NKAT2 has been shown to be a inhibitory KIR that is important in early pregnancy to prevent the attack of trophoblastic cells by NK cells [44]. We found a decreased numbers of NKAT-2^+^ NK cells in SLE patient compared to controls, especially the CD56 bright NK subsets. IL-15 enhanced NKAT-2 expression of CD56 bright NK subsets in SLE patients.

Bai et al showed increased CD158a expression on NK cells from SLE patients [45]. We also observed an increased CD158a expression in CD56^brigtht^ population in SLE compared to controls. A distinct CD158b ^high^ NK subset was found in SLE patients, but not in controls.(Fig 10A), which may contribute to the suppressed NK cytotoxicity observed in SLE patients. IL-15 enhances both CD158a and CD158b expression on NK cells from both SLE patients and controls.

NKAT-2 and CD158b seem to recognize the same group of KIR. However, NKAT-2 was deficient and CD158b was enhanced on NK cells from SLE patients compared to controls. According to the manufacturer’s description, NKAT2 (mAb DX27) recognizes KIR2DL3; while CD158b (mAb CHL) recognizes KIR2DL2, KIR2DL3 and KIR2DS2. Thus, The discrepancy of NKAT2 staining results may be due to their narrower KIR epitope. Some point changes affecting mAb epitopes due to KIR polymorphisms may also influence the results.

CD158k is expressed on a minor population of circulating NK cells [46]. It has not been studied in SLE patients. Unlike NK cells from controls, CD158k on NK cells SLE patients failed to be enhanced by IL-15. Our finding again suggest an IL-15 signaling defect in SLE NK cells.

Taken together, SLE patient showed a distinct NCR and iNKR pattern compared to controls. IL-15 had a heterogeneous effect on NK receptor expression. CD69, CD94, NKG2A, NKp30, and CD158b expression on NK cells from SLE patients were higher than corresponding controls, and could be further enhanced by IL-15. On the other hand, IL-15 enhanced NK cytotoxicity, down-regulated NKP46 expression (which was higher in SLE patients), and enhanced NKG2D and NKAT-2 (which were lower in SLE patients) on NK cells from SLE patients. Therefore, IL-15 could have both disease-promoting and ameliorating effect on SLE. In view of the fact that serum IL-15 was elevated in SLE patient and that IL-15 predominantly aggravates the aberrant NKR expression found in SLE, IL-15 antagonist may have therapeutic benefits in SLE patients. Antagonist to IL-15 may provide a therapeutic option for stopping the progression of SLE.

## Supporting information

S1 Dataset This file depicts the data obtained in the normal control and systemic lupus erythematosis patiens. Headings are depicted in 20170920 Support information.xls.
(XLS)
